# Exploring facilitators and barriers to the implementation of prehabilitation for individuals with pancreatic cancer: a survey of healthcare professionals

**DOI:** 10.1007/s00520-026-11097-9

**Published:** 2026-08-21

**Authors:** Elizabeth Wrench, Elizabeth McGladrigan, Robert M. Lauder, Declan Dunne, Cliff Shelton, Joel Lambert, Laura L. Machin, Daren A. Subar, Theodoros M. Bampouras, Christopher J. Gaffney

**Affiliations:** 1 Lancaster Medical School, Faculty of Health and Medicine, Lancaster University, Lancaster, LA1 4YW UK; 2 School of Sport and Exercise Sciences, Liverpool John Moores University, Liverpool, L3 3AF UK; 3 Blackburn Research Innovation Development Group in General Surgery (BRIDGES), Blackburn, BB2 3HH UK; 4 Department of General Surgery, East Lancashire Hospitals NHS Trust, Blackburn, BB2 3HH UK; 5 Liverpool University Hospitals NHS Foundation Trust, Liverpool, L7 8YE UK; 6 Department of Anaesthesia, Wythenshawe Hospital, Manchester University NHS Foundation Trust, Manchester, M23 9LT UK

**Keywords:** Pancreatic cancer, Prehabilitation, Optimal care pathway

## Abstract

People with pancreatic cancer experience poor survival, low quality of life and limited treatment options compared to those with other cancers; interventions that can improve outcomes and quality of life are urgently required. Pancreatic Cancer UK recently released ‘optimal care guidelines’ for pancreatic cancer, which recommends that all patients, no matter the treatment route, should be offered prehabilitation (typically exercise, nutrition and psychosocial intervention) at the point of diagnosis. We investigated the facilitators and barriers to implementing prehabilitation for individuals with pancreatic cancer, from the perspective of healthcare professionals. A mixed methods cross-sectional survey on the facilitators and barriers to implementing prehabilitation and engaging individuals with pancreatic cancer in prehabilitation was designed and circulated to healthcare professionals (*n* = 138). Responses were analysed using a thematic approach. Key factors highlighted by healthcare professionals that could support the implementation of a prehabilitation intervention included multidisciplinary team cohesion and collaboration and dedicated prehabilitation champions, resources and National Health Service trust engagement, accessibility and avoiding exacerbating health inequalities, community support, and clinical guidelines. Key factors that could overcome barriers to the participation of individuals with pancreatic cancer included educational support, clear aims and information, understanding of the disease-related burden and individualised programmes. Considering each of these factors in the design and delivery of services could inform future research and the implementation of prehabilitation for individuals with pancreatic cancer.

**Supplementary Information:**

The online version contains supplementary material available at https://doi.org/10.1007/s00520-026-11097-9.

## Introduction

Pancreatic cancer is the fourth most frequent cause of cancer-related death, with a 5-year survival rate less than 10% [1]. The only treatment option that offers the potential for a cure is surgery in combination with chemotherapy, yet less than one in ten pancreatic cancer patients in the UK receive surgery [2, 3]. Pancreatic resection is associated with significant morbidity and mortality, and research suggests surgery significantly impacts the quality of life for patients in the postoperative period [4]. Treatments for pancreatic cancer have shown limited progression compared to other cancers, and therefore, interventions which improve both quality of life and outcomes are urgently required [1].

Prehabilitation is predominantly investigated and utilised in the pre-operative period for individuals with cancer. It consists of exercise, nutritional and/or psychological interventions, with a multimodal approach thought to be optimal [5, 6]. Prehabilitation can also be offered prior to other oncological treatments, including chemotherapy and radiotherapy [6]. However, limited research has been conducted on prehabilitation specifically for individuals with pancreatic cancer and it is therefore a key area for further research [7, 8].

**Fig. 1:**
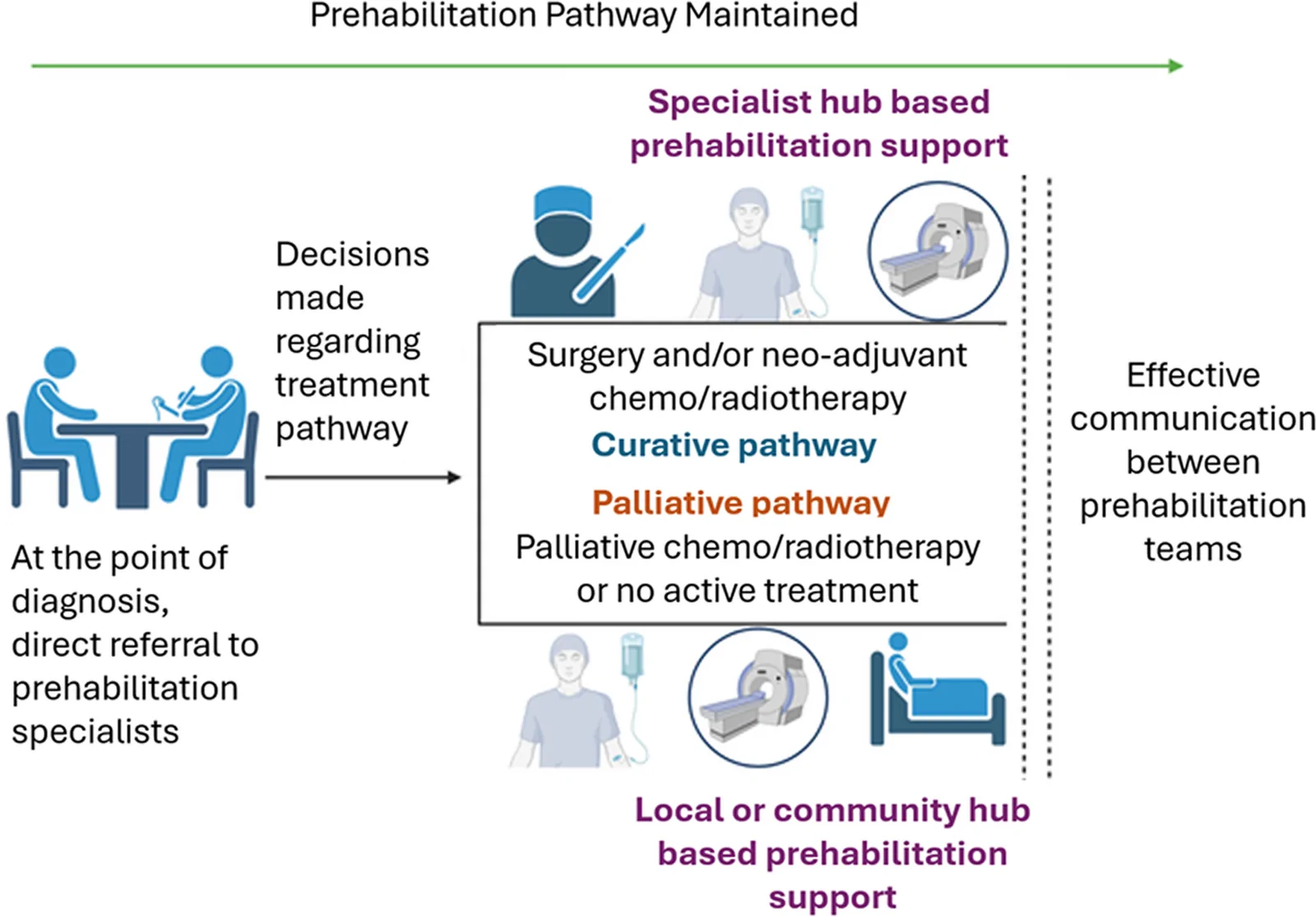
The recommended prehabilitation pathway adapted from Pancreatic Cancer UK’s guidelines [9]. The recommended pathway suggests that all individuals with pancreatic cancer should be offered prehabilitation at the time of diagnosis and referred to the relevant specialists no matter the treatment pathway. This support and effective communication should be maintained between prehabilitation teams if an individual swaps treatment pathways. Created in BioRender. Wrench, L. (2025) https://BioRender.com/t47c130

The optimal care pathway for individuals with pancreatic cancer was published by Pancreatic Cancer UK, recommending all patients with pancreatic cancer should be offered prehabilitation at the point of diagnosis (Fig. 1) [9]. Pancreatic cancer is often associated with a rapid transition from diagnosis to a late palliative phase with severe symptoms and therefore treatment pathways can quickly change [10]. Offering prehabilitation at the point of diagnosis to all patients, prior to decisions regarding treatment, increases the opportunity for all pancreatic cancer patients to benefit, including those on non-surgical pathways. There are a wide range of benefits of prehabilitation, including enhancing patients’ physiological reserve, ensuring appropriate nutrition, reducing length of stay in hospital, attenuating muscle loss and reducing patients’ anxiety and stress, suggesting all pancreatic cancer patients could potentially benefit from this type of intervention [5, 6, 8, 9, 11].

There are challenges with implementing prehabilitation across the NHS, due to a lack of resources and increasing demands [12]. Evidence suggests that prehabilitation could contribute to the widening of health inequalities, with patients having different levels of capacity to engage [13]. Individuals with pancreatic cancer experience additional challenges compared to those with many other cancer types, due to the complexity of the disease and symptoms, the frailty of the patients and poor survival rates [15, 16]. Furthermore, the rapidly progressing nature of pancreatic cancer may necessitate a more adaptive prehabilitation approach to accommodate the individual’s fluctuating symptom burden [10].

Implementation of a prehabilitation programme for individuals with pancreatic cancer is likely to be beneficial but challenging [8]. Therefore, a deeper understanding from the health professionals’ perspective of the facilitators and barriers to both implementation and engagement with a programme is required to inform the development of such a programme. A successful prehabilitation programme is likely to require effective collaboration between key stakeholders, a willingness from staff to engage patients in prehabilitation and patients to be aware of the potential benefits [17, 18]. This study therefore aimed to explore the perspective of healthcare professionals on the facilitators and barriers to implementing and engaging individuals with pancreatic cancer in prehabilitation.

## Methods

### Sample characteristics and ethical approval

Research Ethics Committee approval was obtained from the Lancaster University Faculty of Health and Medicine Research Ethics Committee (FHM-2024-4266-IRAS-1) on 28/02/2024, and Health Research Authority approval was subsequently obtained on 09/04/2024 (24/HRA/1213). To proceed with the survey, individuals had to confirm that they gave informed consent.

The inclusion criteria comprised healthcare professionals who had a role in the care of individuals with pancreatic cancer and worked within NHS trusts or health-related organisations external to the NHS, such as specialist nurses within charities. The survey was hosted on an online platform (Qualtrics, Qualtrics LLC, Utah, USA), circulated via a link that could be accessed anonymously and took approximately 10 minutes to complete. NHS trusts (*n* = 24), listed on Pancreatic Cancer UK’s website as specialist centres (https://www.pancreaticcancer.org.uk/support-for-you/your-care/your-local-pancreatic-cancer-specialist-centre/) and located across England, were contacted and asked to circulate the survey to relevant individuals who might have a role in the care of individuals with pancreatic cancer. Nine NHS trusts were subsequently set up as participant identification centres (PIC). Local collaborators from each PIC were provided information on the study and asked to circulate the survey to individuals who fit the inclusion criteria. Regular discussions were held with local collaborators, and they sent out follow-up emails as necessary.

In addition, healthcare professionals with a publicly available email address and who had a role in the care of individuals with pancreatic cancer were contacted directly by the research team to maximise recruitment. Participants were sampled purposively, but snowball sampling also occurred where healthcare professionals forwarded the survey to relevant colleagues. Nurses or other healthcare professionals working through cancer charities, such as Macmillan, are often integrated within cancer care pathways in the NHS. As a result, contact to participate could have been made by local collaborators, via snowball sampling, or by health-related organisations external to the NHS.

The participant information sheet, including the inclusion criteria, was provided at start of the survey so that all participants had access prior to deciding whether to complete the survey. Individuals were contacted between 11/04/2024 and 31/07/2024. Responses identified as likely to have originated from automated programmes (bots) due to abnormal IP addresses external to the UK and response irregularities were excluded from the dataset after discussion by members of the research team.

### Questionnaire development

The mixed methods cross-sectional survey was designed purposively based on the theoretical domains framework (TDF) as no previously validated survey appropriately answered the research question [19, 20]. The TDF was chosen to develop the survey as it incorporated both behavioural change theory and implementation science into a single framework, both of which need to be considered when understanding the implementation of prehabilitation for individuals with pancreatic cancer [19–21].

The questionnaire topic areas were developed by reviewing previous evidence within the literature on the facilitators and barriers to the implementation of prehabilitation and through discussions within the research team [22, 23]. These topic areas were then mapped and tailored to fit within the TDF. The most relevant domains from the TDF were selected to structure the survey, including knowledge, environmental context and resources, beliefs about capabilities and consequences, social/professional role and identity, social influences, optimism, and intentions [19–21]. The survey was then reviewed and piloted by three subject matter experts who provided detailed feedback on the survey, specifically the content and length, and it was then further refined and tailored to the specific research aims. They helped improve the relevance and content of the survey by refining the number of questions and answers for questions, as well as increasing the clarity of questions asked. These individuals were not excluded from the subsequent data collection. The mapping of questions onto the TDF is shown in Appendix 1.

The survey had four separate sections with 19 questions in total, a combination of both multiple-choice answer questions and three open text responses. The first section of the survey collected demographic characteristics, including the respondent’s NHS trust, the Integrated Care Board (ICB) which oversees the trust, and job role of the individual. A definition of prehabilitation was provided, after which respondents were asked the extent to which they understood the definition of prehabilitation, their familiarity with Pancreatic Cancer UK’s guidelines, and whether they were aware of the recommendations to implement prehabilitation for all individuals with pancreatic cancer [9].

The subsequent sections of the survey had questions focussing on different factors related to healthcare professionals’ views on the facilitators and barriers to implementing prehabilitation for individuals with pancreatic cancer, including attitudes about implementation, resources required, responsibility for implementation and delivery, current availability of services, contextual and environmental facilitators and barriers, and engagement of individuals with pancreatic cancer. A copy of the full survey can be found in Appendix 2.

### Data analysis

Quantitative and qualitative data were analysed separately and merged. The open text responses were analysed by reflexive thematic analysis, following the six stages (familiarisation, coding, generating themes, reviewing themes, defining and naming themes and writing up) as described by Braun and Clarke (2020) [24] to generate key themes around the facilitators and barriers to implementing prehabilitation for individuals with pancreatic cancer. Reflexive thematic analysis was completed iteratively by one member of the research team (EW) to ensure the analysis had a consistent theoretical perspective. Themes were reviewed by EW to ensure coherence and relevance, as well as providing the opportunity to reflect and consider the impact of any assumptions. Once this process was completed, themes were reviewed by three other members of the research team (EM, DAS, TMB), not with the aim of ensuring consensus but to ensure themes accurately reflected the body of evidence and to feedback on relevance and coherence.

Closed text responses were reported using percentages to provide evidence to support the key themes [25]. The descriptive quantitative responses were compared with the generated qualitative themes to understand whether responses aligned or diverged and to determine where qualitative responses could provide depth and understanding to the descriptive quantitative findings. The results of this integration are reported below.

## Results

**Fig. 2:**
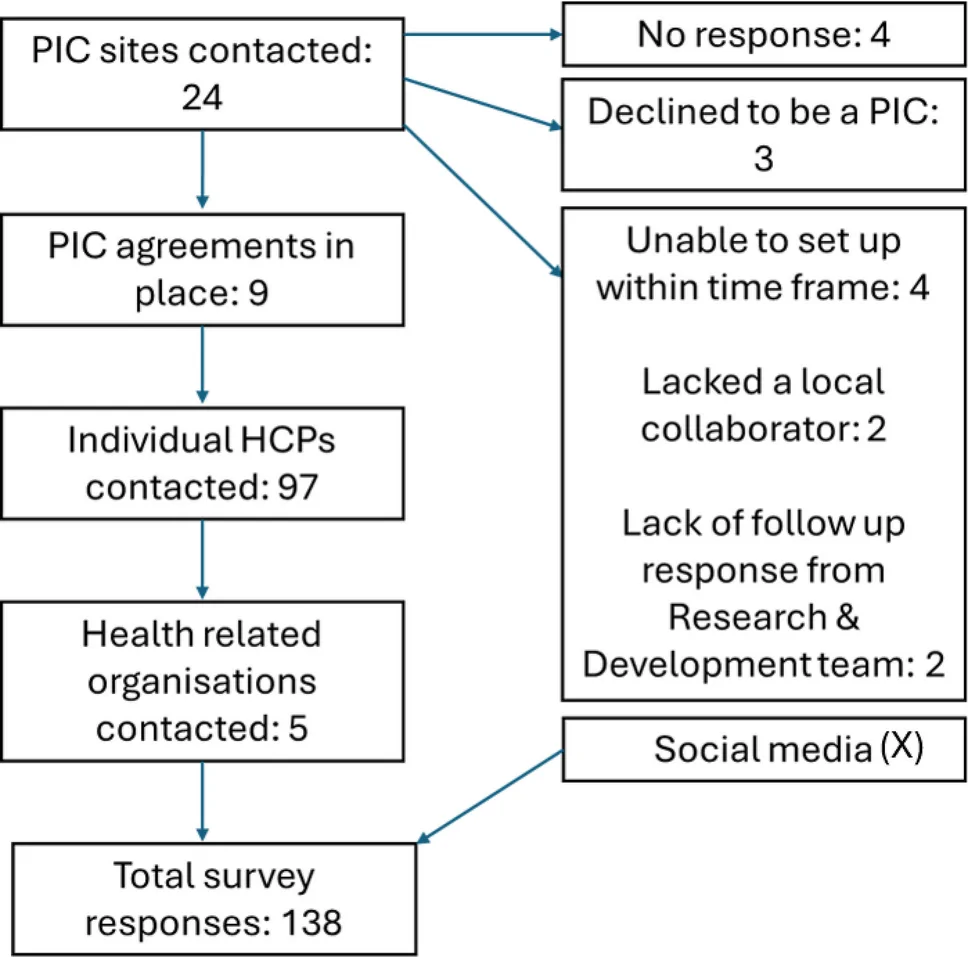
Overview of responses received, from initial contact of NHS trusts and individuals to the final number of responses

Through publicly available email addresses, 97 healthcare professionals were contacted individually, in addition to those contacted by NHS trusts and recruited from the social media platform X, as shown in Fig. 2. In total, 138 survey responses were received from healthcare professionals based within 24 ICBs, verifying the data collected had national reach. Of the 138 survey responses, 123 participants finished the survey and the lowest number of respondents to a closed text question was 118. Results are therefore presented as both percentages and counts out of the number of responses to specific questions. As shown in Table 1, healthcare professionals with varied job roles responded to the survey improving the representation of the sample for a range of healthcare professionals involved in the care of individuals with pancreatic cancer.

**Table 1:** The job roles of healthcare professionals that responded to the survey. Data is presented as counts and percentage (in brackets) of the overall sample (*n* = 138)

Job role	Count (%)
HPB/pancreatic cancer nurse specialist	38 (27.5%)
General surgeon	11 (8.0%)
Dietitian	20 (14.5%)
Oncologist	14 (10.1%)
Gastroenterologist	6 (4.3%)
Physiotherapist	8 (5.8%)
Palliative care nurse	2 (1.4%)
HPB surgeon	11 (8.0%)
Clinical research nurse/practitioner	15 (10.9%)
Other	13 (9.4%)

A large proportion of healthcare professionals (106/132, 80%) reported being familiar with Pancreatic Cancer UK’s guidelines recommending the implementation of prehabilitation for individuals with pancreatic cancer. Further, 56% (74/131) strongly agreed, 24% (31/131) somewhat agreed whereas 17% (22/131) strongly disagreed with the recommendation.

However, when filtering the results to just the 17% of healthcare professionals that strongly disagreed with implementing prehabilitation for all individuals with pancreatic cancer, 100% (22/22) of those believed that it was important, 59% (13/22) believed it would be useful and 9% (2/22) believed it would be stressful for individuals with pancreatic cancer. Some evidence within the open text responses suggests not ‘all’ patients may benefit. In particular, it was highlighted that *“Patients need to be fit enough as this would not be appropriate in patients where best supportive care is the recommendation”* (DoWI). This could therefore be a potential reason why some people strongly disagree that prehabilitation should be implemented for all individuals with pancreatic cancer but still believe it is important.

### Who or what else is needed to successfully implement prehabilitation (facilitators)?

The questions which contributed to the generation of the following themes focussed on understanding who should be responsible for the implementation of prehabilitation, what resources are required and what are the contextual and environmental facilitators and barriers which would impact implementation of a programme for individuals with pancreatic cancer.

#### MDT cohesion and collaboration

**Fig. 3:**
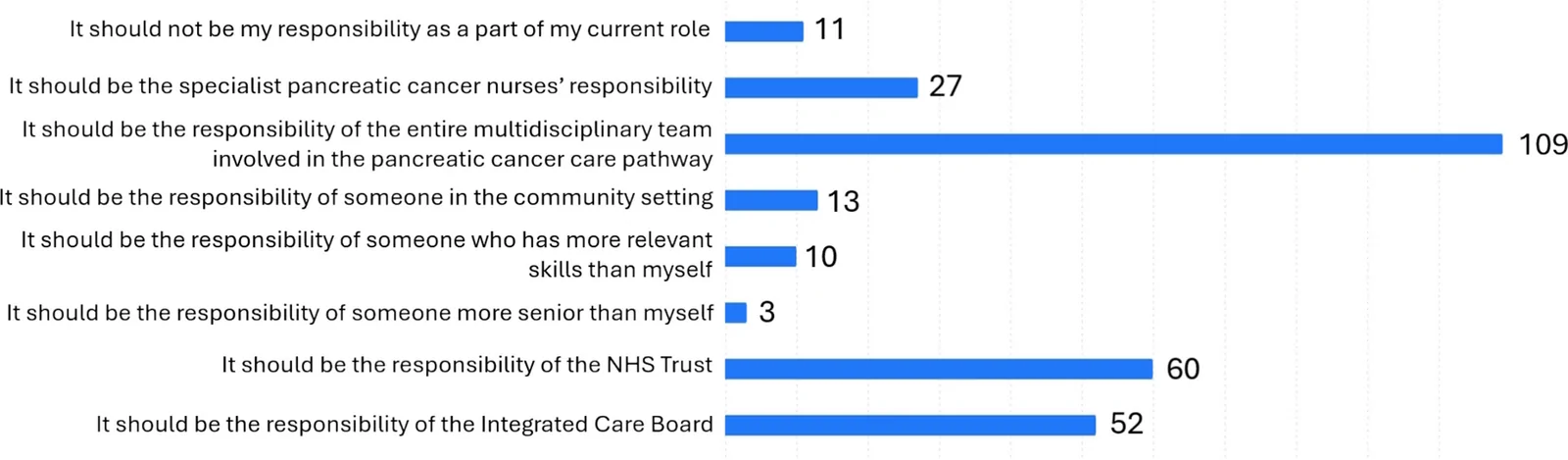
Whose responsibility should it be to organise prehabilitation for individuals with pancreatic cancer?

As shown in Fig. 3, healthcare professionals were largely congruent regarding the responsibility for the organisation and implementation of prehabilitation for individuals with pancreatic cancer, with 85% (109/128) responding it should be the responsibility of the entire multidisciplinary team (MDT). Effective MDT communication and collaboration between all healthcare professionals involved in the care of individuals with pancreatic cancer was also highlighted as key to ensuring the effective implementation of prehabilitation by the following quotes:

*‘A MDT approach with physios, dietitians and a specific prehab nurse in order to co-ordinate and push this.’ (U1wd)*

*‘Multidisciplinary working and embedding prehab as part of the patient pathway’ (72mi)*

#### Resource constraints and NHS trust engagement

**Fig. 4:**
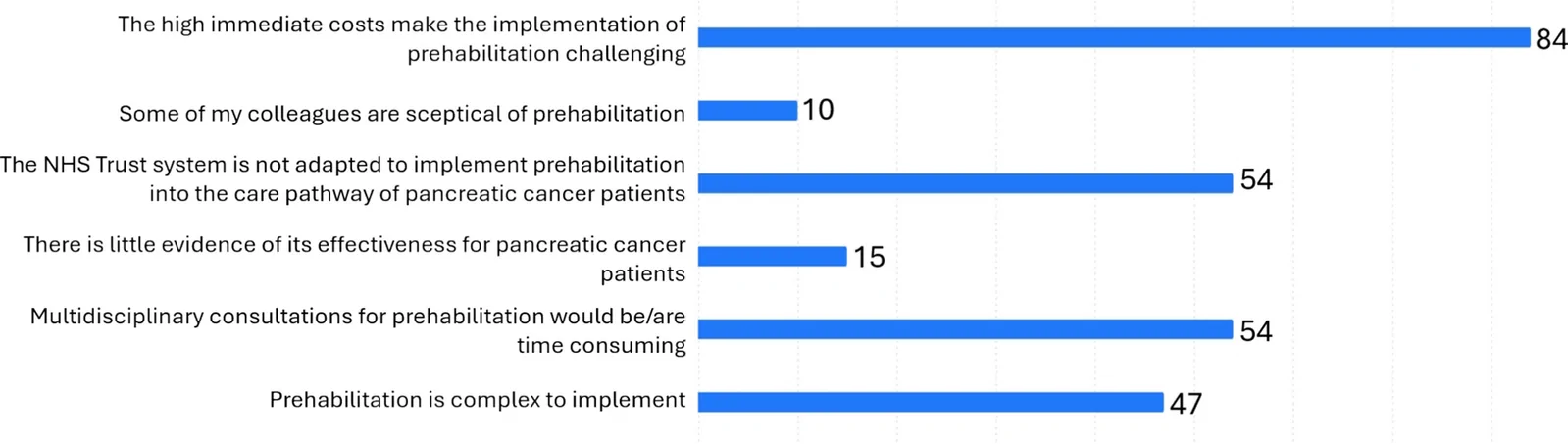
What are the contextual and environmental barriers to organising prehabilitation for individuals with pancreatic cancer?

Resources were highlighted as one of the biggest barriers with 71% (84/118) of respondents stating that the high immediate costs would make the implementation of prehabilitation challenging (Fig. 4), particularly for funding staff time or additional staff to provide specialist support when implementing prehabilitation for these individuals. NHS trust engagement and support was highlighted as a key facilitator and 47% (60/128) of respondents said that organising prehabilitation should be the responsibility of the NHS trust. Resource constraints and NHS trust engagement were also highlighted within the open text responses:

*‘Funding to enable access to appropriate staff (dietician (we have no Oncology/Upper GI dietician), physio, exercise programme staff, psychology or counselling support)’ (5EZ3)*

*‘Investment. We do not have the expertise, equipment, staff or time to implement this - we can't even get timely dietetic advice for cachectic patients!’ (vVrl)*

*‘funding in our Trust!! To ensure multimodal prehab can be given. Support from entire MDT as well as board directors in Trust’ (z8Dk)*

#### Dedicated prehabilitation champions

Respondents identified that dedicated prehabilitation specialists or champions within the trust *"a champion/specialist within this tumour group would be helpful"* (Mzyk) to advocate for the importance of prehabilitation for individuals with pancreatic cancer were important facilitators for programme success. Eighty-four percent (101/120) of healthcare professionals who are/or would be involved in prehabilitation agreed they should be provided with specific training.

*‘Every trust should have dedicated prehabilitation team with full training and knowledge with good support from all the necessary specialities to deliver successful prehabilitation program’ (jczP)*

#### Accessibility and not exacerbating health inequalities

Healthcare professionals highlighted the importance of ensuring implemented programmes do not exacerbate health inequalities by making it challenging to access and engage with the intervention. It was also highlighted that there should be *"equitable access across all pathways"* (VPcK) so that all pancreatic cancer patients, *"surgical and non surgical"* (VPcK), should be given the opportunity of prehabilitation.

*‘Often our patients are not local and struggle/cannot be expected to attend face-to-face for all of their prehab appointments.’ (fWvx)*

*‘lack of access/understanding of technology (for virtual sessions). Lack of family/friend support. Lack of transport - hospital transport takes hours out their day. Lack of self-efficacy.’ (32J2)*

#### Community support

Healthcare professionals suggested that providing support within the local community and being able to utilise local facilities where patients could be referred could help reduce the burden on their workload and help ensure the programme is accessible, especially the exercise aspect. Qualitative evidence highlighted that *"NHS teams should promote the concept/provide details on where classes can be accessed" (i9aN)* and "*Support of leisure centres to allow patients to access exercise locally (for a certain period of time)" (32J2)* could be a facilitator*.*

#### Clinical guidelines

**Fig. 5:**
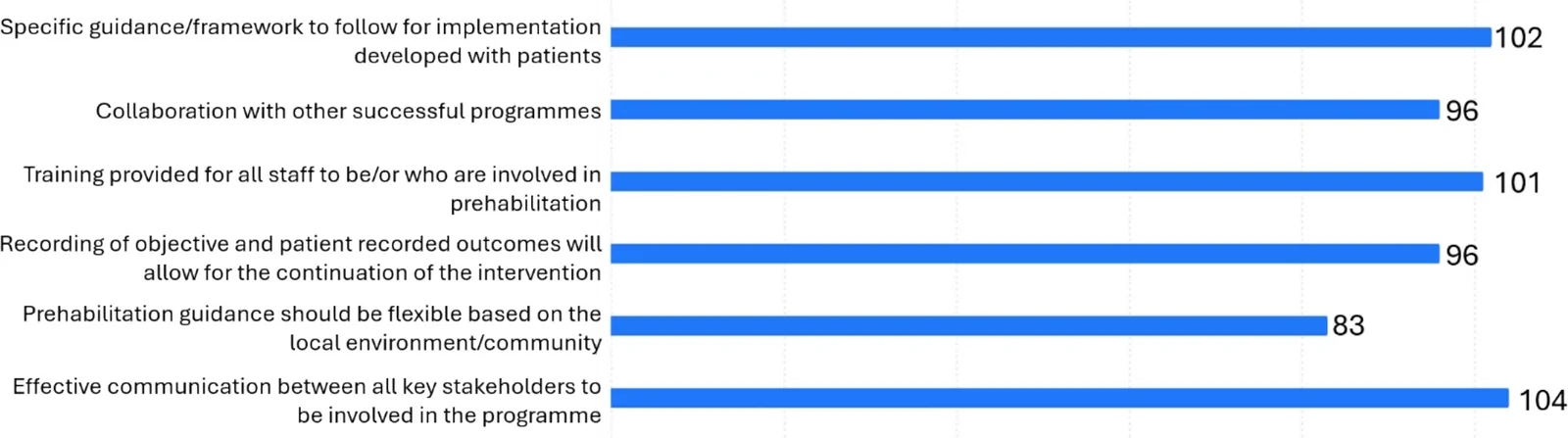
What are the contextual and environmental factors that would facilitate prehabilitation (select all that apply)?

Respondents suggested a clear need for a common framework or clinical guidelines which NHS trusts can follow to support the implementation of a programme, as shown in Fig. 5. Eighty-five percent (102/120) of healthcare professionals felt that specific guidance/advice to follow for implementation and developed alongside patients would be a key factor facilitating prehabilitation. Qualitative evidence highlighted that *"there are no structured guidelines to follow" (CE1m)* and prehabilitation for individuals with pancreatic cancer "*Needs adoption into clinical guidelines, otherwise it will not be implemented. Budgetary considerations, scope *etc." (pt5v). In addition, another open text response which supported clinical guidelines as a facilitator to implementation suggested *"Not having a common framework within the different trust involved in the patients care"* (VLKQ) currently acts as a barrier. Eighty percent (96/120) of respondents also felt it would be useful to collaborate with programmes that have been successfully implemented for individuals with pancreatic cancer.

### From the perspective of healthcare professionals, what facilitators and barriers would impact the engagement of individuals with pancreatic cancer with prehabilitation?

The questions which were used to generate the below themes asked healthcare professionals to consider their patients and identify what difficulties individuals with pancreatic cancer might face when engaging with a programme and what could help facilitate their participation.

#### Concerns and disagreement over increasing patient burden

**Fig. 6:**
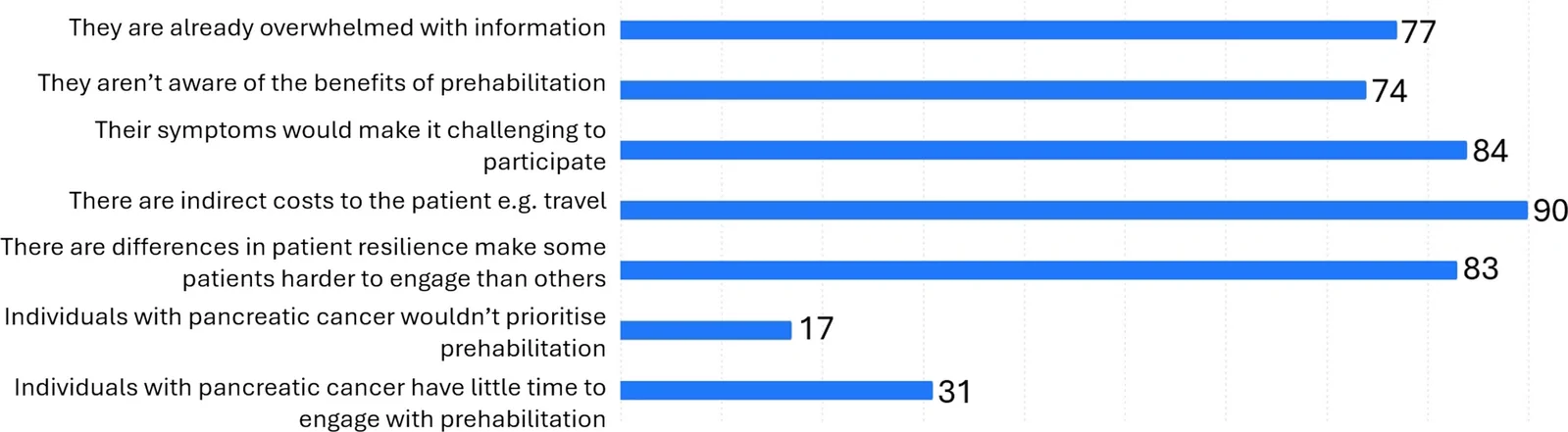
It would be difficult to engage individuals with pancreatic cancer because … (select all that apply)

As shown in Fig. 6, several healthcare professionals highlighted concerns over the symptoms (70%, 84/120) and information overwhelm (64%, 77/120) experienced by individuals with pancreatic cancer which would make it challenging for the patients to engage in prehabilitation. This was supported by more detail in the qualitative responses *"The emotional impact of a pancreatic cancer diagnosis means you cannot speak to them about prehab at their diagnosis appointment"* (U1wd) and *"Overwhelming amount of information. treatment side effects. lack of resources. difficulty in changing perception of dietary advice for these patients"* (gT3U). Thirteen percent (15/118) of healthcare professionals said there is little evidence on the effectiveness within this patient group.

*‘Our biggest issues in this patient group are that pancreatic patients are often referred to perioperative care very late in their pathway, they live miles away from the hospital and are often the most unwell meaning they really struggle to do any meaningful amount of exercise prehab.’ (sic) (lyol)*

In contrast, 8% (10/118) of respondents said that some of their colleagues are sceptical of prehabilitation for individuals with pancreatic cancer and the open text responses highlighted some common misconceptions and beliefs among healthcare professionals that patients are too ill or would not benefit from a prehabilitation intervention.

*‘Main barrier is professionals’ perception and being ill-informed that pancreatic patients won’t benefit from prehab as too unwell/or irreversible condition’ (duJw)*

*‘Pre-existing barriers including misconceptions about the disease group (pancreatic cancer) would potentially impact on the programme’ (SzYJ)*

These concerns therefore suggest that further research is required to develop understanding and an evidence base on the effectiveness and feasibility of prehabilitation specifically for individuals with pancreatic cancer.

#### Patient education

Sixty-two percent of (74/120) healthcare professionals highlighted that patients were not aware of the benefits of prehabilitation which would act as a barrier to engagement and *"More time then needs to be spent on education and building rapport"* (PWxj). Patient education could therefore be a vital facilitator between patients and engagement, and this was highlighted in one of the themes arising from the open text box responses*, "A key facilitator would be to inform patients of the benefits to their health and outcomes from engagement with prehab" (ANeh).*

#### Empowering patient participation

Eighty-six percent (104/121) of healthcare professionals believed that empowering patients to play an active role in their own treatment would be beneficial. Eighty percent (97/121) of healthcare professionals believed that individualised programmes were key and 82% (99/121) also believed that specific goals and targets would facilitate engagement within prehabilitation. This is supported by one of the key themes within the open text responses in terms of empowering patients to participate.

*‘explicit goals and milestones to reach, and clear alternatives offered’ (uKNh)*

*‘Range of setting; virtual, group, flexible dates & times etc.’ (mHgj)*

*‘some patients may feel intimidated at the thought of working out.’ (s8cW)*

#### Disease-related burden

Seventy percent (84/120) of healthcare professionals believed the symptoms individuals with pancreatic cancer experience would make it challenging to participate in prehabilitation. This was a key theme highlighted by the open text responses regarding the facilitators and barriers to engaging patients.

*‘Patients who present acutely late in their diagnosis may not have the energy to participate’ (ETvl)*

*‘physical issues such as fatigue or sickness’ (FZrH)*

*‘Adjustment depression disorder will be quite common among newly diagnosed patients so psychological support/counselling’ (E6Zz)*

#### Understanding patient priorities

Another key theme that arose during the open text responses was the recognition that participation in prehabilitation may not be the individual patient’s priority. This needs to be considered when approaching individuals with pancreatic cancer about the programme.

*‘Patient may feel their time is precious and not want to return to hospital for regular pre-hab appointments.’ (RVvo)*

*‘For inoperable patients they have a lot of symptoms that will affect their ability to engage fully with a programme. They also want to focus on QoL [quality of life] and time with families/friends rather than another appt [appointment] at the hospital.’ (u1wd)*

#### Participation ‘opt-out’ system

Eighty-seven percent (105/121) of healthcare professionals thought the implementation of prehabilitation early in the trajectory of care for patients would facilitate engagement, with qualitative evidence suggesting *"Really embedding it into the pathway would help ie it is not optional and is bought into by the whole MDT [multidisciplinary team]"* (Mzyk). Additionally, 88% (106/121) of respondents felt that prehabilitation appointments should be combined with other appointments to happen on the same day. Healthcare professionals suggested in the open text box response that an opt-out programme might be useful to ensure patients get referred onto the programme at the start of their care pathway* "Dedicated prehabilitation teams and general support of these teams. At [trust] prehab is an “opt-out” programme" (JU1q).*

## Discussion

**Fig. 7:**
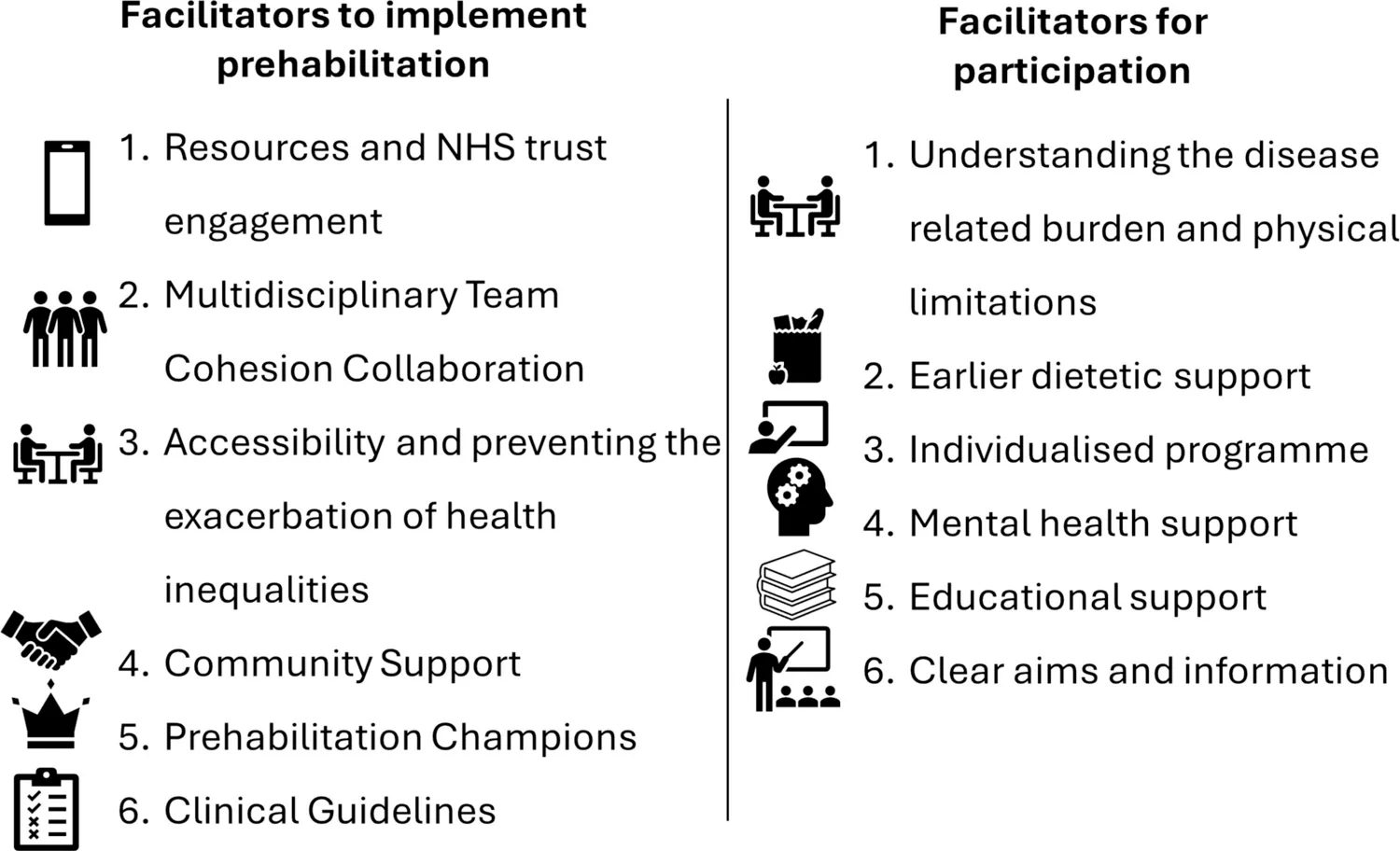
The key facilitators to the implementation of prehabilitation and engaging with individuals with pancreatic cancer in a prehabilitation programme

Prehabilitation, traditionally implemented prior to surgery, has increased momentum to be adopted across the cancer care pathway to improve patient outcomes prior to treatment [26]. Pancreatic cancer, however, presents unique challenges, particularly related to the specific symptom burden and rapid progression that must be considered when deciding how to implement prehabilitation [27]. This study therefore focussed on assessing the perspective of healthcare professionals on implementing and engaging all individuals with pancreatic cancer in prehabilitation, with the key findings from the study highlighted in Fig. 7.

### What are the facilitators for engaging individuals with pancreatic cancer in prehabilitation?

#### Patient education and understanding informational needs

In general, prehabilitation has shown to improve functional capacity, reduce the length of hospital stays and also improve health-related quality of life, all of which could potentially benefit individuals with pancreatic cancer [6, 11]. One of the key facilitators highlighted by healthcare professionals was educating individuals with pancreatic cancer and healthcare professionals on the potential benefits of prehabilitation or its components such as nutrition and exercise. Continuing professional development or eLearning specifically focussing on prehabilitation for individuals with pancreatic cancer could help educate healthcare professionals on some of the potential benefits of prehabilitation so they can be better informed when having discussions with patients.

From the perspective of healthcare professionals, the findings from this study suggest that patient education, additional resources and clear objectives of prehabilitation could address unmet informational needs and allow patients to make more informed decisions about their lifestyle and prehabilitation, hopefully reducing barriers to engagement with a programme. Evidence within the literature, from the perspective of patients, suggests that more than one quarter of individuals with pancreatic cancer have unmet needs regarding the information provided at the point of diagnosis [28]. The largest informational needs for individuals with pancreatic cancer were identified as psychological, such as uncertainty, fear and depression, and physical factors such as fatigue, along with those related to daily living and being unable to complete activities they conducted pre-diagnosis [28]. The unmet informational needs highlighted by patients within the literature and the perspectives highlighted by healthcare professionals within this study suggest there are specific gaps in patient education that prehabilitation offered to all individuals with pancreatic cancer at the point of diagnosis could potentially help to fulfil.

Healthcare professionals believed that empowering individuals with pancreatic cancer to play an active role in their own treatment would be beneficial and having clear goals and milestones could increase engagement. Evidence suggests that individuals with advanced cancer often experience a loss of control, and this loss of control can be associated with increased distress and negative outcomes [29]. However, being able to make informed choices around lifestyle, wellbeing and treatment can help an individual maintain some control and improve perceived quality of life [29]. Qualitative evidence collected on individuals undergoing chemotherapy for oesophagogastric cancer and participating in prehabilitation identified similarly that attainable goals and objectives helped increase motivation as well as adherence with a prehabilitation programme [30]. The time and format in which the information should be provided requires further research.

#### Consideration of the specific disease-related burden of pancreatic cancer

Healthcare professionals highlighted that factors relating to an individual’s symptoms would make it challenging to participate in prehabilitation. These factors commonly related to energy, anxiety, and sickness, aligning with those discussed in the literature [31, 32]. This highlights the likely need for prehabilitation to be personalised rather than standardised with healthcare professionals believing that individualised programmes would be a key facilitator to engagement.

Some healthcare professionals in this study also highlighted concerns that certain patients may not be offered prehabilitation based on their perceived capabilities and potential misconceptions of the disease group. Research on the priorities of patients found that individuals with pancreatic cancer prioritised physical function and their ability to carry out daily physical and social activities whereas healthcare professionals prioritised ‘pain, anxiety and depression’ symptoms to be targeted [33]. This suggests that the implementation of prehabilitation could help meet the priorities of individuals with pancreatic cancer to improve daily function and further research is required to investigate this. For it to be successful, the implementation of prehabilitation is likely to benefit from public and patient co-production, to ensure that prehabilitation meets patients’ needs rather than just the healthcare professionals’ perception of what their needs may be.

### What are the facilitators for the effective implementation of a prehabilitation intervention for individuals with pancreatic cancer?

#### Resources and NHS trust engagement

Healthcare professionals were posed with the specific question as to who or what else was required to successfully implement prehabilitation for individuals with pancreatic cancer. A substantial proportion of responses related to funding and, more specifically, funding for additional staff or time. Some evidence within the literature suggests prehabilitation, specifically pre-operative prehabilitation, could be cost-effective when compared to ‘standard’ care, for example by reducing length of stay in hospital post-surgery [34]. However, evidence is lacking for pancreatic cancer prehabilitation. This suggests further research is warranted into the optimal programme and cost-effectiveness of prehabilitation specifically in pancreatic cancer. This would assist in constructing business cases for funding that aligns with the principles of value-based healthcare [35, 36].

Implementation of a prehabilitation programme specifically for individuals with pancreatic cancer within an NHS trust is also likely to require high-level organisational engagement and support, along with the buy-in of external stakeholders. This could help overcome some of the barriers faced regarding limited resources and coordination of a programme. Due to the rapid deterioration of the disease, individuals with pancreatic cancer may have a rapidly changing care pathway, depending on their current disease stage, which is likely to require high-level effective coordination to implement a programme [9, 10]. To facilitate trust engagement, further research needs to be carried out on the benefits of prehabilitation in all individuals with pancreatic cancer with objective outcomes reported and the potential cost-effectiveness of a programme to be completely understood. This information would help to inform a detailed business case, as described in the literature [37, 38].

#### Ensuring accessibility and preventing the exacerbation of health inequalities

Specific factors, such as transport and technology, were discussed as barriers to participation, along with challenges some patients might face, for example lack of transport to travel to an in-person programme or the lack of technology to access an online programme, need careful consideration, especially when preference for prehabilitation delivery could depend on the patient’s current symptom burden and family support available. This is increasingly important considering the NHS highlighted within its National Healthcare Inequalities Improvement Programme that restoration of inclusivity in NHS services and preventing digital exclusion should be prioritised [39]. The optimal method of prehabilitation delivery would require research to be undertaken.

Another key factor highlighted by respondents was ensuring the accessibility of prehabilitation for all patients. It was noted that in several pre-existing prehabilitation programmes, prehabilitation was not accessible to all patients, particularly individuals with pancreatic cancer but only individuals with certain diseases or cancer types at specific points of their patient pathway, often due to a lack of funding and capacity. This could also be influenced by limited evidence on the impact of prehabilitation for individuals with pancreatic cancer, indicating the need for further research.

#### Community support

One theme that arose from healthcare professionals was support within the community, which could be provided by collaborating with local leisure centres. Collaboration with local leisure facilities has already proven successful in large-scale prehabilitation interventions such as Prehab4Cancer in Greater Manchester which had strong partnerships with the external stakeholders [40]. Using a similar model for individuals with pancreatic cancer could reduce the distance required for patients to travel, reduce the burden placed on NHS staff to provide this arm of the intervention, and create a support group of individuals going through the same thing as cancer peer support groups have proven to have a number of benefits on individuals’ wellbeing [41].

Home-based prehabilitation has also shown to be successful as a delivery method with high adherence to programmes; however, research needs to be conducted specifically on those with pancreatic cancer [42]. Delivery of a programme through technology within an individual’s home might overcome some of the concerns regarding patients who do not have access to transport, who live a significant distance from the hospital providing treatment, or who would find travel difficult, for example because of the fluctuating and rapidly deteriorating symptom burden associated with pancreatic cancer [10]. Further investigation into the best method of delivery is required with significant input from individuals with pancreatic cancer and their family members.

#### Multidisciplinary team cohesion and collaboration and dedicated prehabilitation champions

Effective working of the entire MDT was identified as a clear theme which could impact the success of the implementation of a prehabilitation intervention for individuals with pancreatic cancer. The MDT brings together a range of expertise to decide and deliver a patient’s care, allowing for more efficient communication, collaboration, and problem-solving [43]. Evidence in the literature suggests that the success of a prehabilitation programme could rely on the integration and effective working of the MDT [44]. This could be particularly important for individuals with pancreatic cancer who may have a rapidly changing care pathway linked to the rapid deterioration of the disease and therefore are transferred between a number of different specialities for appropriate care [9, 10].

A clinical champion working within the MDT and specialising in prehabilitation for individuals with pancreatic cancer would help advocate and support prehabilitation in this patient group [45]. They could be involved in a range of ways, such as training and education of staff members and patients, encouraging engagement and buy-in, and also promoting positivity about the intervention [45]. Clinical champions play a critical role in many implementation strategies [45, 46]. Indeed, prehabilitation champions were identified as a key facilitator by healthcare professionals within this survey and therefore should be considered in implementation strategies for prehabilitation for individuals with pancreatic cancer.

#### Clinical guidelines

Eighty-five percent of healthcare professionals believed that specific guidance to follow would be a key facilitator. Clinical guidelines are used to help standardise and enforce best practice across healthcare by taking into consideration a wide evidence-base, cost-effectiveness, and professional and patient expertise [47]. Prehabilitation for all pancreatic cancer patients is already acknowledged as a priority in the Optimal Care Pathway of Pancreatic Cancer UK and Macmillan have recently released clinical and implementation guidelines for prehabilitation for people with cancer [9, 14]. However, to be adopted by trusts in the UK, guidance is required from the National Institute for Health and Care Excellence (NICE) in a specific set of clinical guidelines to follow [47]. This would likely necessitate further research in this specific patient group to develop the evidence base along with health professionals and health-related organisations external to the NHS, to advocate for the guidelines to be produced.

### Could an opt-out intervention serve as an effective intervention strategy?

Time, the appropriate moment to approach the patient, increasing health inequalities, and effective coordination were all discussed as facilitators and barriers within this study. One suggestion was to introduce an opt-out prehabilitation programme, and this was highlighted as already being in place at one NHS trust in England. This would mean patients are automatically referred into the programme with the decision regarding whether to opt out left for them to decide.

Having a default option for prehabilitation is an example of a choice architecture intervention whereby an individual is directed or nudged towards the desirable option of participating in prehabilitation whilst maintaining their own freedom of choice to ‘opt-out’ [48]. A streamlined process such as this could help improve effective coordination, reduce concerns regarding when to approach the patient, reduce the time spent deciding whether to refer a patient, and reduce the risk of health inequalities if all patients are at least referred to the service. For individuals with pancreatic cancer, automatic enrolment into a prehabilitation programme could also potentially reduce decision fatigue and information overwhelm at a time when they are likely to have a substantial number of decisions to make [49]. Limited research is available on opt-out prehabilitation programmes within the literature and, as such, this is an important area for future research.

## Limitations

The survey for healthcare professionals aimed to reach as many different healthcare professionals as possible. The representativeness of respondents was carefully monitored and purposive sampling of clinical specialities with low response rates was conducted to try and maximise representativeness of the participating healthcare professional population. Every effort was made to advertise the survey as open to all individuals with a role in the care of pancreatic cancer patients. The main respondents were clinical nurse specialists, general surgeons, dietitians, and oncologists, and we had a limited number of physiotherapists, palliative care specialists, and clinical psychologists take part in the survey. As a result, the findings of this report are not generalisable to all clinical specialities involved in the care of individuals with pancreatic cancer. Prehabilitation is traditionally seen as a pre-operative intervention to improve surgical outcomes, and therefore, those that do not have a role traditionally in prehabilitation may have been less likely to respond. As a result, the survey results could be biased towards those that already play an active part in prehabilitation or have a keen interest in prehabilitation, and this must be taken into consideration when interpreting the results.

Healthcare professionals were encouraged to circulate the survey to any other individuals who fit the recruitment criteria. As a result of this snowball sampling, individuals interested in prehabilitation may have been more likely to respond, impacting the external validity of the results, and this should be taken into consideration when interpreting the results. Further research is required to understand the perspectives of those under-represented in this research, such as palliative care professionals.

## Conclusions

This study collected the opinions of healthcare professionals to understand potential facilitators and barriers to the implementation and engagement with a prehabilitation intervention for all individuals with pancreatic cancer. Key factors were highlighted from a healthcare professional perspective which could improve the effectiveness of implementation, along with facilitators for individuals with pancreatic cancer to participate, as shown in Fig. 7. Facilitators include factors such as MDT cohesion, NHS trust engagement, consideration of the disease-related burden, education, and clear aims and outcomes. Consideration of an opt-out intervention could help mitigate and streamline the implementation and referral of patients to prehabilitation. Further research is required to determine the optimal delivery of prehabilitation for individuals with pancreatic cancer and the effectiveness in this specific patient group.

## Supplementary Information

Below is the link to the electronic supplementary material.

ESM 1 (DOCX 253 KB)

ESM 2 (DOCX 25.8 KB)

## Data Availability

The participants of this study did not give written consent for their data to be shared publicly, so due to the sensitive nature of the research supporting data is not available.
