# Supplementary material for: Exploring facilitators and barriers to the implementation of prehabilitation for individuals with pancreatic cancer: a survey of healthcare professionals
Source: Support Care Cancer. 2026 Aug 21;34(9):883. doi: 10.1007/s00520-026-11097-9 (PMC13498508; doi:10.1007/s00520-026-11097-9)
Supplement: Supplementary file 1 [file 520_2026_11097_MOESM1_ESM.docx]

**Appendix 1** – Mapping of the questionnaire onto the domains of the Theoretical Domains Framework
