# Supplementary material for: Exploring facilitators and barriers to the implementation of prehabilitation for individuals with pancreatic cancer: a survey of healthcare professionals
Source: Support Care Cancer. 2026 Aug 21;34(9):883. doi: 10.1007/s00520-026-11097-9 (PMC13498508; doi:10.1007/s00520-026-11097-9)
Supplement: Supplementary file 2 [file 520_2026_11097_MOESM2_ESM.docx]

Appendix 1 – The survey questions circulated to healthcare professionals.

Please tell us about yourself:

Q1 Place of work:

________________________________________________________________

Q2 Job role:

- Pancreatic cancer nurse specialist
- General surgeon
- Dietician
- Oncologist
- GP
- Radiotherapist
- Gastroenterologist
- Physiotherapist
- Occupational Therapist
- District or community nurse
- Palliative care nurse
- Counsellor
- Clinical psychologist
- Other
- *If other please state __________________________________________________

Q3 Where is your role based?

- Community
- Hospital
- Charity or organisation exernal to the NHS

Q4 Which ICB does your place of work belong to?

- NHS Bedfordshire, Luton and Milton Keynes ICB
- NHS Cambridgeshire and Peterborough ICB
- NHS Hertfordshire and West Essex ICB
- NHS Mid and South Essex ICB
- NHS Norfolk and Waveney ICB
- NHS Suffolk and North East Essex ICB
- NHS North Central London ICB
- NHS North East London ICB
- NHS North West London ICB
- NHS South East London ICB
- NHS South West London ICB
- NHS Birmingham and Solihull ICB
- NHS Black Country ICB
- NHS Coventry and Warwickshire ICB
- NHS Derby and Derbyshire ICB
- NHS Herefordshire and Worcestershire ICB
- NHS Leicester, Leicestershire and Rutland ICB
- NHS Lincolnshire ICB
- NHS Northamptonshire ICB
- NHS Nottingham and Nottinghamshire ICB
- NHS Shropshire, Telford and Wrekin ICB
- NHS Staffordshire and Stoke-on-Trent ICB
- NHS Humber and North Yorkshire ICB
- NHS North East and North Cumbria ICB
- NHS South Yorkshire ICB
- NHS West Yorkshire ICB
- NHS Cheshire and Merseyside ICB
- NHS Greater Manchester ICB
- NHS Lancashire and South Cumbria ICB
- NHS Buckinghamshire, Oxfordshire and Berkshire West ICB
- NHS Frimley ICB
- NHS Hampshire and Isle of Wight ICB
- NHS Kent and Medway ICB
- NHS Surrey Heartlands ICB
- NHS Sussex ICB
- NHS Bath and North East Somerset, Swindon and Wiltshire ICB
- NHS Bristol, North Somerset and South Gloucestershire ICB
- NHS Cornwall and The Isles Of Scilly ICB
- NHS Devon ICB
- NHS Dorset ICB
- NHS Gloucestershire ICB
- NHS Somerset ICB

Q5 I am familiar with the recommendation from Pancreatic Cancer UK’s optimal care pathway that prehabilitation should be available to all pancreatic cancer patients.

- Yes
- No

Q6 I agree with Pancreatic Cancer UK’s guidelines to implement prehabilitation for all pancreatic cancer patients.

- Strongly disagree
- Somewhat disagree
- Somewhat agree
- Strongly agree

What is prehabilitation?
Prehabilitation enables people with cancer to prepare for treatment or improve quality of life through promoting healthy behaviours and through needs-based prescribing of exercise, nutrition and psychological interventions. (Adapted from Macmillan)

Q7 To what extent do you understand prehabilitation as defined above?

- I completely understand prehabilitation as described above.
- I somewhat understand prehabilitation as defined above.
- I do not understand prehabilitation as defined above.

Q8 What are your beliefs about prehabilitation? (select all that apply)

- I believe it is important for individuals with pancreatic cancer.
- I believe it is useful for individuals with pancreatic cancer.
- I believe prehabilitation would be stressful for individuals with pancreatic cancer.
- I don’t believe individuals with pancreatic cancer will benefit from prehabilitation.
- I believe prehabilitation could improve the quality of life of individuals with pancreatic cancer.

Q9 What resources do you feel are required to implement prehabilitation for pancreatic cancer patients? (Select all that apply)

- Specific training
- Protected time within the role of individuals required for prehabilitation
- Financial support to implement a programme
- A prehabilitation coordinator
- Specific equipment eg exercise equipment
- Dedicated space

Q10 Whose responsibility should it be to organise prehabilitation for individuals with pancreatic cancer? (select all that apply)

- It should not be my responsibility as a part of my current role.
- It should be the specialist pancreatic cancer nurse’s responsibility.
- It should be the responsibility of the entire multidisciplinary team involved in the pancreatic cancer care pathway.
- It should the responsibility of someone within a community setting.
- It should be the responsibility of someone who has more relevant skills than myself.
- It should be the responsibility of someone more senior than myself.
- It should be the responsibility of the NHS trust
- It should be the responsibility of the integrated care board (ICB)

Q11 Has prehabilitation already been implemented or do you intend to implement prehabilitation?

- We already offer multimodal prehabilitation
- We already offer single mode nutritional prehabilitation
- We already offer single mode exercise related prehabilitation
- We already offer single mode psychosocial related prehabilitation
- We tried to implement prehabilitation but it was unsuccessful, if so why was it unsuccessful? __________________________________________________
- I don’t think prehabilitation will be useful to implement in individuals with pancreatic cancer, if so why? __________________________________________________
- We don't currently offer prehabilitation for individuals with pancreatic cancer, but I would like to see it implemented

Q12 Who or what else is required to **successfully implement** a prehabilitation programme for individuals with pancreatic cancer?

________________________________________________________________

Q13 What are the contextual and environmental **barriers** to organising prehabilitation for individuals with pancreatic patient? (select all that apply)

- The high immediate costs make the implementation of prehabilitation challenging.
- Some of my colleagues are sceptical of prehabilitation.
- The NHS trust’s system is not adapted to implement prehabilitation into the care pathway of pancreatic cancer patients.
- There is little evidence on its effectiveness for pancreatic cancer patients.
- Multidisciplinary consultations for prehabilitation would be/are time consuming.
- Prehabilitation is complex to implement.

Q14 What are the contextual and environmental factors that would **facilitate** prehabilitation? (select all that apply)

- Specific guidance/framework to follow for implementation developed with patients.
- Collaboration with other successful prehabilitation programmes.
- Training provided for all staff to be/or who are involved in prehabilitation.
- Recording of objective and patient recorded outcomes will allow for the continuation of the intervention.
- Prehabilitation guidance should be flexible based on the local environment/community.
- Effective communication between all key stakeholders to be involved in the programme.

Q15 How would other facilitators and barriers impact a prehabilitation programme being implemented for pancreatic cancer patients?

________________________________________________________________

Thinking about your patients:

Q16 Considering operable and inoperable patients with pancreatic cancer, who do you think would benefit from prehabilitation? (select all that apply)

- Operable patients would benefit from prehabilitation prior to surgery
- Inoperable patients would benefit from prehabilitation prior/during treatment
- Best supportive care patients (inoperable patients not planning to undergo any treatment) would benefit from prehabilitation/rehabilitation to improve quality of life

Q17 It would be difficult to engage individuals with pancreatic cancer because ….. (select all that apply)

- They are already overwhelmed with information
- They aren’t aware of the benefits of prehabilitation
- Their symptoms would make it challenging to participate
- There are indirect costs to the patient eg, travel
- There are differences in patient resilience making some patients harder to engage than others
- Individuals with pancreatic cancer wouldn’t prioritise prehabilitation.
- Individuals with pancreatic cancer have little time to engage with prehabilitation

Q18 What would **facilitate** patient engagement in prehabilitation?

- Individualised programmes
- Combining prehabilitation appointments with other appointments to happen on a single day
- Offering an intervention close to home
- Offering an intervention remotely
- Implementing prehabilitation early in the trajectory of care
- Set specific goals which can be used to motivate patients
- Personal support from family members during prehabilitation
- Group activities to exchange experiences and motivate each other.
- Empowering patients to play an active role in their own care.

Q19 How might other barriers and facilitators impact patients **engaging** with prehabilitation?

________________________________________________________________
